# A Variant Quorum Sensing System in *Aeromonas veronii* MTCC 3249

**DOI:** 10.3390/s120403814

**Published:** 2012-03-26

**Authors:** Kamlesh Jangid, Perunninakulath S. Parameswaran, Yogesh S. Shouche

**Affiliations:** 1 Microbial Culture Collection, National Centre for Cell Science, Pune, Maharashtra 411007, India; E-Mail: yogesh@nccs.res.in; 2 National Institute of Oceanography, Dr. Salim Ali Road, P.O. Box 1913, Kochi, Kerala 682018, India; E-Mail: param@nio.org

**Keywords:** *Aeromonas*, *luxRI* homolog, *acuR*, *acuI*, quorum sensing, AHL, mosquito midgut, 6-carboxy-HHL

## Abstract

We have investigated the quorum sensing control in *Aeromonas veronii* MTCC 3249, originally isolated as *A. culicicola* from the midgut of *Culex quinquefasciatus.* Based on biosensor assays, the bacterium showed constant production of multiple acyl-homoserine lactones (AHLs) with increasing cell-density. The *luxRI* gene homologs, *acuR* (*A. culicicola* transcriptional Regulator) and *acuI* (*A. culicicola* autoInducer) were successfully amplified by inverse-PCR. Sequence analysis indicated *acuRI* were divergent from all known quorum sensing gene homologs in *Aeromonas*. Two localized regions in the C-terminal autoinducer binding domain of *acuR* showed indels suggesting variations in autoinducer specificity. Further, only a single copy of the quorum sensing genes was detected, suggesting a tight regulation of mechanisms under its control. Chromatography and further chemical analysis identified two AHLs in the culture supernatant: 6-carboxy-HHL (homoadipyl homoserine lactone), a novel AHL, and *N*-tetradecanoylhomoserine lactone. The existence of a potentially variant quorum sensing system might therefore, reflect in some way the ecological strategies adopted by this bacterium in the mosquito midgut.

## 1. Introduction

*Aeromonas* spp. are important disease-causing pathogens of fish and other cold-blooded species, as well as humans [1]. Many of its virulence determinants are expressed at high cell densities in the late exponential phase and may thus be under quorum sensing control [2]. Further, the close proximity of LuxRI gene homologs to *iciA*, an inhibitor of chromosome replication links quorum sensing and cell division in this genus. Recently, homologs of the *V. fischeri luxRI* genes have been detected in most *Aeromonas* species [3], unlike a few years ago, when only two species, *A. hydrophila* and *A. salmonicida*, were known to secrete quorum sensing molecules.

The discovery of quorum sensing in *Aeromonas* has placed focus on this genus for the elucidation of its role in pathogenesis. Unlike *A. hydrophila*, wherein both the serine and metallo protease activities are under quorum sensing control [4], the general secretory pathway for the export of serine protease in *A. salmonicida* (*exe*) is not under quorum sensing control [2]. Further, the production of extracellular proteases in *A. hydrophila* is decreased in the presence of long chain N-acyl-L-homoserine lactones (AHLs) such as 3-oxo-C_12_-HSL. Thus, the quorum sensing system could be a potential target for novel pharmaceutical agents based on the long chain AHLs to decrease the virulence of the bacterium [4].

The increasing incidences of isolation of *Aeromonas* species from both clinical and environmental samples have interested microbiologists worldwide. One such strain, *A. culicicola* MTCC 3249, later determined to be a subjective synonym of *A. veronii* [5], was isolated from the midgut of *Culex quinquefasciatus* by Pidiyar *et al.* [6]. In a study on the effect of *A. veronii* MTCC 3249 on the susceptibility of *Culex quinquefasciatus* to the Japanese encephalitis virus (JEV), the bacterium increased the susceptibility of mosquitoes to JEV when added in the mosquito's blood meal [7]. Further, 27 strains of *A. veronii* were isolated from drinking water supply in Spain [8] suggesting a diverse worldwide distribution of the species. Given that contaminated drinking water supply is a major source of diarrhoeal diseases in Spain, the pathogenic potential of this species must be tested.

In addition, gaining an insight into the quorum sensing machinery of *A. veronii* MTCC 3249 has become a priority. This hemolytic strain was reported to rapidly increase its cell number (2,000-fold) after the blood meal of mosquito [6]. Similar observations were reported in other species of *Aeromonas* that form biofilms and inhabit nutrient rich, localized environments [2,4,9,10]. The rapid growth of midgut bacteria after blood meal may be fuelled by the iron and protein-rich bolus of blood [11]. However, such high increase in cell number also suggests the presence of a very efficient regulatory mechanism. Given the close proximity of *iciA* homolog downstream of the autoinducer synthase gene in *Aeromonas*, the involvement of quorum sensing in increasing the cell density cannot be ruled out. This tempted us to investigate the nature of this control.

We also investigated whether *A. veronii* contained paralogs of the *acuRI* quorum sensing genes. We hypothesized that like many other bacteria, such as *Clostridium perfringens* [12], *Erwinia carotovora* [13], *Pseudomonas aureofaciens* [14], *etc., A. veronii* MTCC 3249 might also possess multiple quorum sensing systems. This stems from the fact that *luxRI* gene homologs in *Aeromonas* share high sequence similarity [3] and it is likely that if multiple quorum sensing systems are present in this strain, these might also share high sequence similarity with the *acuRI* system. Although this specific aspect has not been investigated in the genus *Aeromonas*, results from *A. hydrophila* and *A. salmonicida* [2,4] suggest the involvement of multiple AHLs and as-yet unidentified factors controlling different phenotypes under quorum sensing control. Hence, the existence of multiple quorum sensing systems cannot be ruled out. Here, we present an analysis of the quorum sensing system in *A. veronii* MTCC 3249 that might in some way reflect on its ecological strategies in mosquito midgut.

## 2. Experimental Section

### 2.1. Bacterial Strains, Plasmids, Media, and Culture Conditions

*A. hydrophila* ATCC 7066^T^, *A. veronii* MTCC 3249, AHL responsive biosensor strain *Chromobacterium violaceum* CV026 [15] and *E. coli* JM109 harboring plasmids pSB401 and pSB403 [16] and pJBA89 [17] were maintained on Luria-Bertani (LB) medium throughout the study and incubations were carried out at 30 °C. Wherever required, the medium was supplemented with ampicillin (50 μg/mL) or kanamycin (40 μg/mL) or tetracycline (20 μg/mL). Cell growth was monitored by measurement of the optical density of culture medium at 540 nm.

### 2.2. Growth Dependent AHL Production by *A. veronii* MTCC 3249

The production of AHLs by *A. veronii* MTCC 3249 was detected using the recombinant plasmid pJBA89 in *E. coli* JM109 as described previously [3]. To determine the critical cell density at which *A. veronii* shows a rapid increase in the production of the AHLs, a growth phase dependent induction of bioluminescence by AHLs in sterile culture filtrate was studied as described previously [18]. Total viable count of *A. veronii* were achieved by spread plating 100 μL aliquots of different dilutions in duplicates at various time intervals during the growth in LB at 30 °C at 150 rpm. For bioluminescence induction, 100 μL supernatant fractions collected at various time intervals upon centrifugation of culture for 3 min at 12,000 rpm was mixed in a 96-well plate with 100 μL of the recombinant *E. coli* JM109 containing pSB401 diluted to an optical density of 0.8 at 450 nm and incubated for 30 min. Bioluminescence counts were taken on Top Count NXT, a microplate scintillation and luminescence counter (Packard, Germany). Fold induction was calculated by substracting the counts of negative control (LB with *E. coli* pSB401) from the entire test samples and then dividing each sample value with the zero minute count. The positive control included 1 μM final concentration of HHL. All experiments were repeated twice and final readings are an average of the two.

### 2.3. DNA Isolation, PCR/Inverse PCR Amplification, Hybridization and Sequencing

Genomic DNA was isolated from the two *Aeromonas* strains using the standard phenol/chloroform/ isoamyl alcohol method [19]. To PCR amplify the *luxRI* gene homologs from *A. veronii*, primers were designed using GeneRunner v3.01 (www.generunner.net) based on *A. hydrophila* (X89469) and *A. salmonicida* (U65741) *luxRI* gene homolog sequences. All primers were numbered according to the 3′ end binding site on the *ahyRI* gene (Table 1). PCR conditions used were: initial denaturation at 95 °C for 3 min; 35 cycles of denaturation at 95 °C for 1 min, annealing at 50 °C for 1 min and extension at 72 °C for 1 min; and a final extension at 72 °C for 10 min. PCR amplicons were purified by PEG/NaCl precipitation and sequenced in-house on an ABI-3730 automated DNA analyzer.

**Figure 1.**
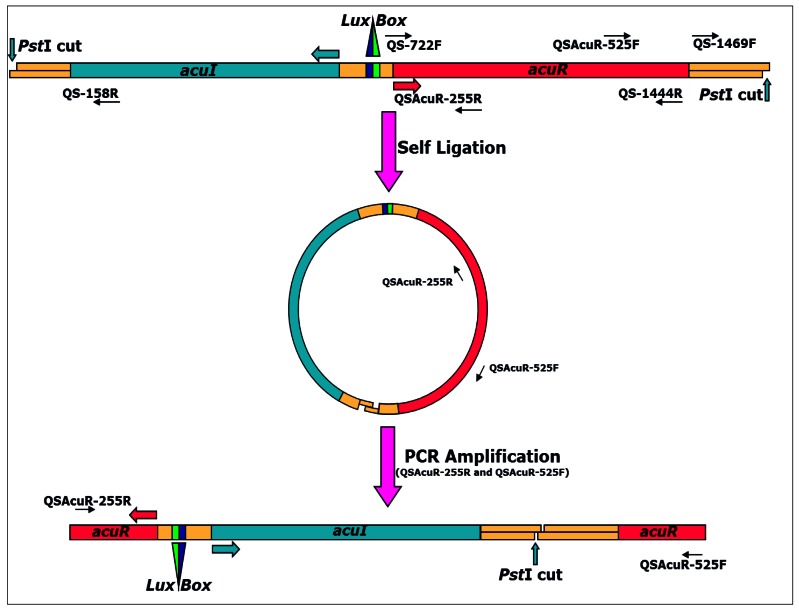
Strategy for Inverse PCR amplification of *acuRI* from *A. veronii*.

For Inverse PCR amplification of the flanking regions, the strategy depicted in Figure 1 was used. All the enzymatic manipulations were carried out as described previously [19] or wherever necessary as described by the manufacturer. Briefly, 10 μg of *A. veronii* genomic DNA was digested with 10 U of the restriction enzyme in 1× digestion buffer in 25 μL reaction volume at 37 °C for 12–14 h. Upon digestion, heat denaturation was done at 80 °C for 15 min and 2 μL of the digested fragments was checked on 0.8% agarose in 0.5× TBE. The remaining digested product was purified using the QIAQuick PCR purification kit (QIAGEN) according to manufacturer's instructions.

Purified DNA fragments were self-ligated in 10 μL reaction volume containing 5 μL of purified product, 1× ligation buffer and 20 U of the T4 DNA ligase at 16 °C for 16 h. Upon ligation, 2 μL was used for a 50 μL PCR reaction using primers QS*AcuR*-255R and QS*AcuR*-525F under similar conditions as described above except that annealing was at 60 °C and cycle elongation was at 72 °C. Amplicons (10 μL) were run against 1 kb plus DNA marker (Invitrogen, USA) in 1% agarose gel in 1× TBE buffer at 5 V/cm for 4 h and then processed for southern hybridization to target the fragment containing the gene of interest. All protocols for blot preparation, probing, hybridization and developing were carried out essentially as described previously [19]. Blots were prepared on Hybond N^+^ paper (Amersham Pharmacia, USA) after depurination, denaturation and neutralization treatments followed by ∼4 h transfer in 20× SSC using vacuum blotting apparatus. Upon transfer, the blot was washed gently in 2× SSC, air dried and processed for hybridization at 55 °C as described previously [5]. PCR product amplified using primers QS*AcuR*-525F and QS-1444R was used as the probe.

DNA fragments corresponding to the putative *acuRI* genes detected in Southern hybridization were eluted from the remaining 40 μL amplicons run on 1% agarose gel in 1× TAE (pH = 8.0) under similar conditions. Elution was carried out using the QIAquick gel extraction kit (QIAGEN) according to manufacturer's instructions. The eluted fragments were cloned in pGEM-T Easy vector (Promega, USA) at molar ratios of 1:3 for vector:insert in a final reaction volume of 5 μL and transformed in *E. coli* JM109 competent cells as described previously [20]. LB Agar plates supplemented with ampicillin (100 μg/mL), X-gal (40 μg/mL) and IPTG (0.1 mM) were used for plating 100 μL transformed culture suspension. Clones were picked after 12–14 h incubation at 37 °C and screened by direct-colony PCR using vector specific PUC1 and PUC2 primers under the conditions described above for inverse PCR. Amplicons from positive clones were purified using PEG/NaCl precipitation and sequenced as above. Nucleotide sequence of the complete genes along with its flanking regions is submitted to GenBank with accession number AY989817.

### 2.4. Determination of *acuRI* Copy Number

Southern hybridization for *acuRI* copy number was performed as described previously for *rrn* operon [21]. PCR amplified *acuR* gene from *A. veronii* using primers *AcuR*F and *AcuR*R was used as the probe. Amplicons were purified by PEG-NaCl precipitation, and random labelled using Megaprime DNA labelling system (Amersham Pharmacia Biotech UK Ltd.). The PCR conditions used were as described above except that annealing was at 55 °C. Hybridization was performed at 60 °C for 14 h in a solution containing 5× SSC, 0.5% SDS, 5× Denhardt's solution, 0.2 mg of denatured salmon sperm DNA mL^−1^ and 2 ng of radiolabeled probe/mL at a specific activity of >1 × 10^8^ dpm/μg.

### 2.5. Chemical Characterization of AHLs Produced by *A. veronii* MTCC 3249

For thin-layer chromatography (TLC) detection of AHLs, 5 mL of *A. veronii* culture supernatant was extracted three times with dichloromethane (7:3 supernatant/dichloromethane). The dried extract was reconstituted in 50 μL HPLC grade acetonitrile and 5 μL samples were subjected to analytical TLC on C_18_ reverse-phase chromatography plates (catalogue no. 4801 425; Whatman), using 60% (vol/vol) methanol in water as the mobile phase as described previously [22]. AHLs were identified by overlaying the chromatograms with a thin layer of LB agar (45 mL) seeded with CV026 (5 mL overnight culture). Plates were incubated at 30 °C overnight and examined for purple spots. Synthetic AHL standards applied in 2 μL volume onto the plates as a common mixture of BHL (catalogue no. 09945; Fluka), HHL (catalogue no. 09926; Fluka), OHL (catalogue no. 10940; Fluka), DHL (catalogue no. 17248; Fluka) and dDHL (catalogue no. 17247; Fluka) were used for reference.

Putative AHLs were extracted and purified from 12 L of stationary-phase *A. veronii* culture grown in M9 medium (SIGMA, USA) with 0.2% acid hydrolyzed casein (Oxoid) as described previously [2]. Extracts were vaccum dried on a Büchi rotavapor R-200 (Büchi Labortechnik, Switzerland), reconstituted in acetonitrile, and then subjected to analytical TLC (as above) and preparative HPLC (as following). Fractions were separated using Supelco PLC8 (250 by 21.2 mm) column (Chromeleon, DIONEX Corporation, USA) with an isocratic mobile phase of 70% (vol/vol) acetonitrile in water at a flow rate of 2 mL per min and monitored at 210 nm. Fractions showing activity in the CV026 reporter assay were pooled and re-chromatographed by using 60% (vol/vol) acetonitrile in water; the procedure was repeated, using a final chromatographic separation employing 30% (vol/vol) acetonitrile in water. Active fractions with same retention times were pooled and analyzed by MS and NMR.

## 3. Results and Discussion

### 3.1. Production of AHLs by *A. veronii* MTCC 3249

**Figure 2.**
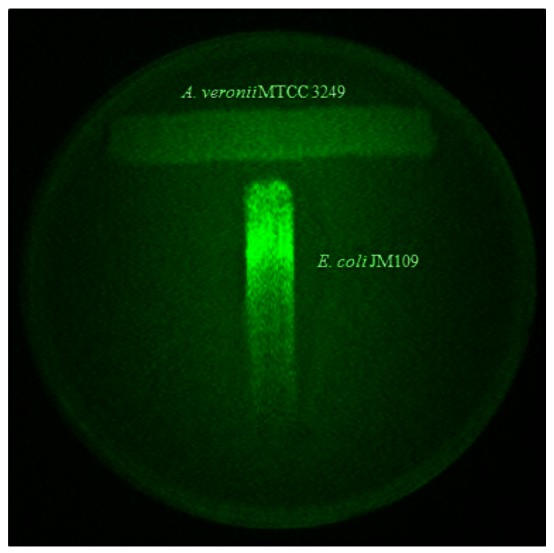
Expression of *gfp* by *E. coli* JM109 pJBA89 in response to AHL production by *A. veronii* MTCC 3249.

**Figure 3.**
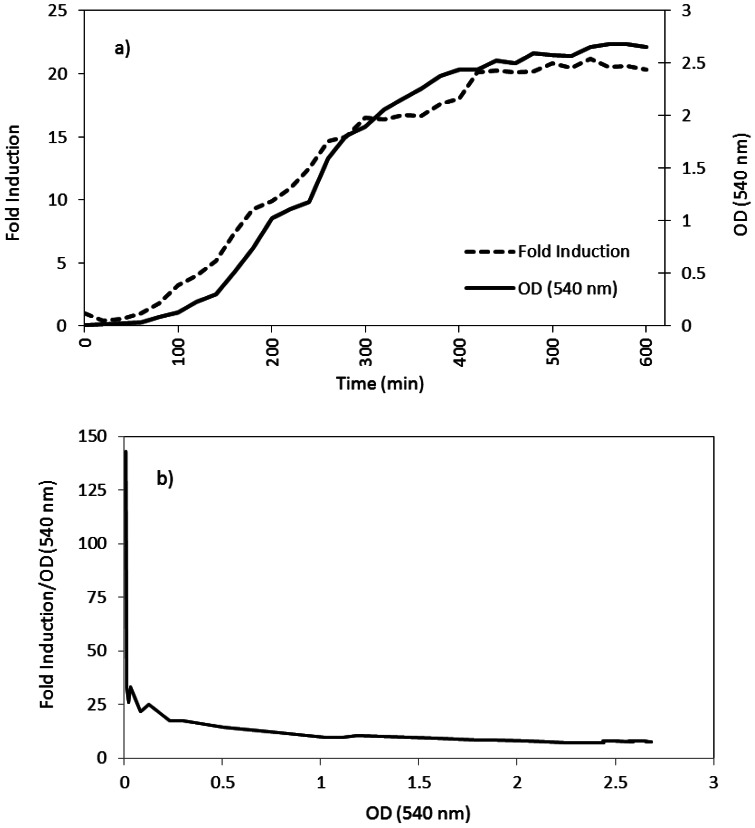
Growth dependent AHL production by *A. veronii* MTCC 3249. (**a**) Fold induction of bioluminescence in *E. coli* JM109 with pSB401 as a function of increasing AHL concentration in the culture supernatant; (**b**) Constant production of AHLs by *A. veronii* in the culture supernatant proportional to its CFU/mL.

Sensitive detection of AHLs secreted by *A. veronii* was achieved by using recombinant derivatives of *E. coli* containing genes expressed only in the presence of AHLs and measured quantitatively. While *A. veronii* MTCC 3249 has already been shown to possess LuxRI gene homologs [3], the bioassay based detection reconfirmed the corresponding phenotype in this strain (Figure 2). The classical “T” shows the decreased *gfp* expression by the indicator strain as a function of the diffusion of the compound in the medium. The production of AHLs was highly correlated with increasing cell density of *A. veronii* (Figure 3(a)). A curve with zero slope was observed when fold induction/OD540 was plotted (Figure 3(b)) indicating that the production of AHLs was constant and proportional to CFU/mL of *A. veronii* MTCC 3249, and there was a linear correlation between AHL amounts and production of light by *E. coli* JM109 with pSB401. The slight differences could be minor variations due to the experimental error of measurements.

### 3.2. *A. veronii* MTCC 3249 *acuRI* System

Based on the sequence analysis of *ahyRI* and *asaRI*, eight primer pairs were designed to amplify the corresponding LuxRI gene homologs from *A. veronii*. However, only one pair, QS-722F and QS-1444R amplified a single DNA fragment (∼790 bp), determined to be the homolog of LuxR- type transcriptional regulator protein was annotated as *acuR* (for *A. culicicola* Regulatory protein).

The flanking regions of the *acuR* gene were amplified by Inverse PCR (Figure 1). Of the four restriction enzymes: *Bgl*II, *EcoR*I, *Hind*III and *Pst*I, chosen on the basis of absence of recognition sites in both *acuR* and *ahyRI/asaRI* sequences, only *Pst*I digest of *A. veronii* MTCC 3249 yielded a single band. The region corresponding to *luxI* homolog was annotated as *acuI* (for *A. culicicola* autoInducer synthase protein).

Both *acuR* and *acuI* shared very low sequence similarity with previously known *luxRI* homologs in *Aeromonas*. Thus, *acuR* shared only 85–87% sequence similarity with *ahyR* and *asaR*, whereas the latter two shared 99% sequence similarity between them. Similarly, *acuI* shared only 70–71% sequence similarity with *ahyI* and *asaI*, whereas the latter two were 88% similar. This low similarity probably explains the failure of attempts to get the *acuI* gene fragment using degenerate PCR and heterologous southern hybridization.

**Figure 4.**
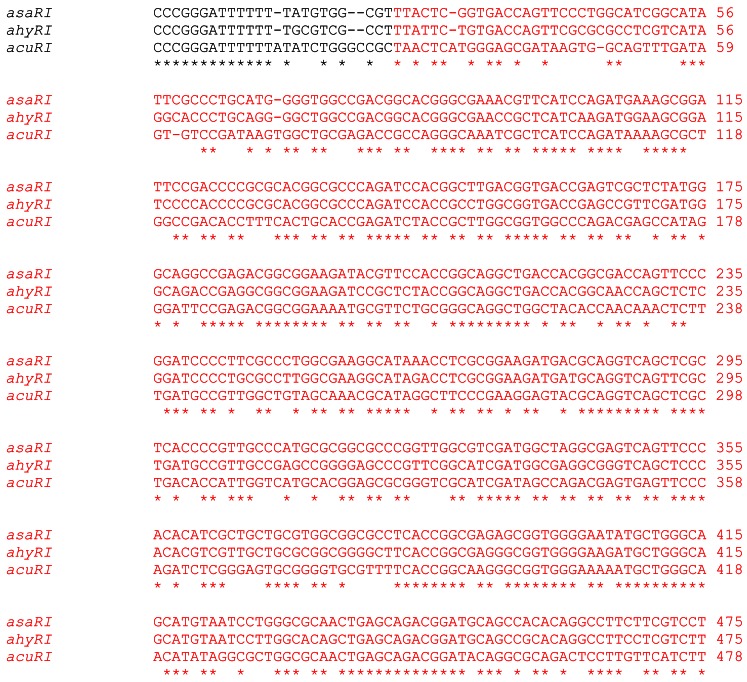

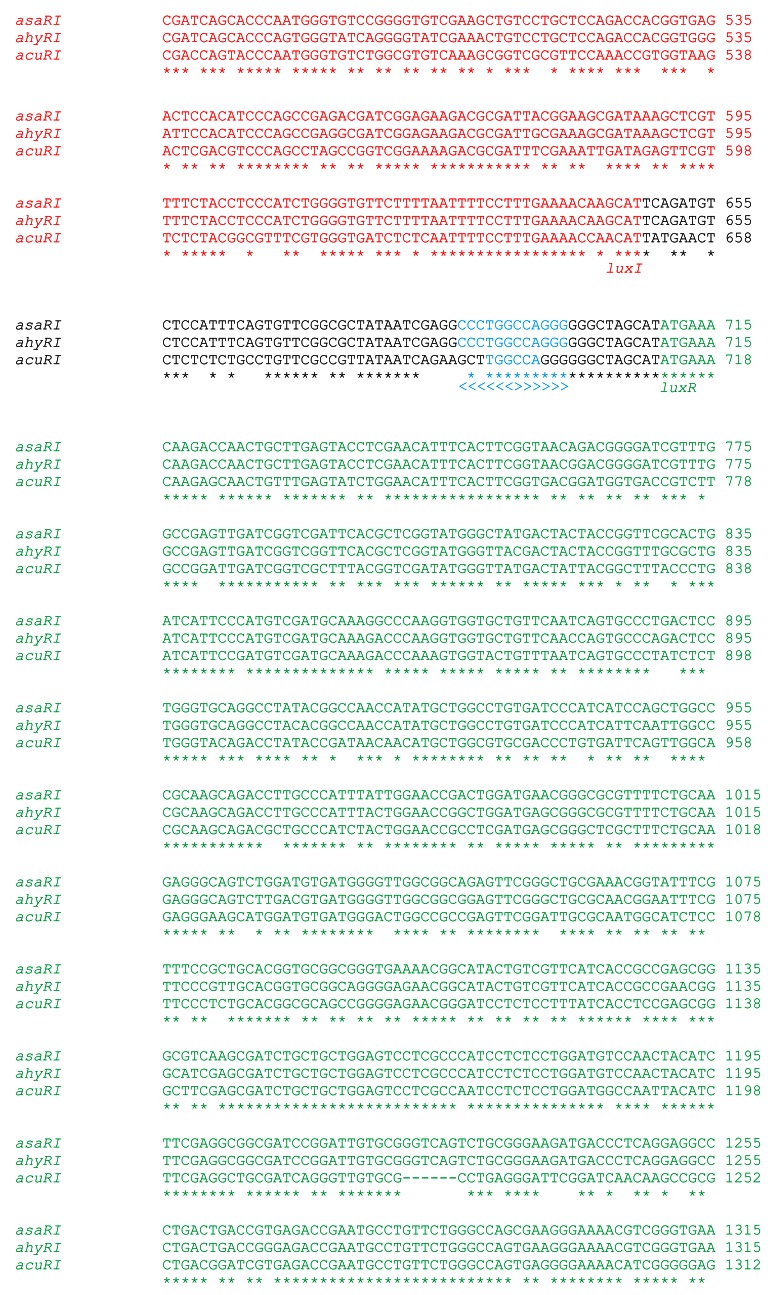

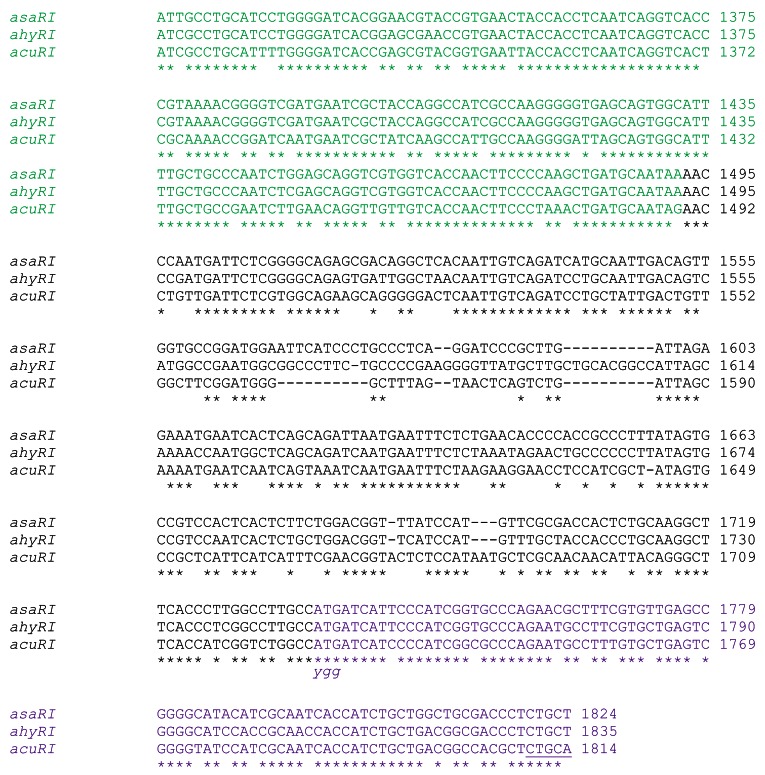
*luxRI* homolog sequences of *A. hydrophila* (*ahyRI*, X89469), *A. salmonicida* (*asaRI*, U65741), and *A. veronii* (*acuRI*, AY989817). *luxI* homologs (red), *luxR* homologs (green), and *ygg* homolog (purple). Region of dyad symmetry, ><, not homologous to the *lux* box consensus sequence (blue region). *Pst*I site (underlined).

In addition to the low similarity, the *acuRI* gene sequence showed unique features suggesting a variant quorum sensing system in *A. veronii*. Specifically, a 6 nt deletion in *acuR* (position 1225), an insertion in *acuI* (position 32), two separate deletions in *acuI* (positions 48 and 61) and a shorter *lux* box region were present in the *acuRI* gene sequence (Figure 4). These indels were important because the insertion lied in the primer binding site of the degenerate primers and justified the inability to PCR amplify the *acuI* fragment in the first step. Further analysis of *acuR* sequence at NCBI's Conserved Domain Search (CD Search) confirmed that the 6 nt deletion lied in the C-terminal binding domain of the general family of transcriptional regulators. This domain contains a helix-turn-helix motif and binds DNA and thus, the deletion may be important, although this has not been investigated yet.

**Figure 5.**
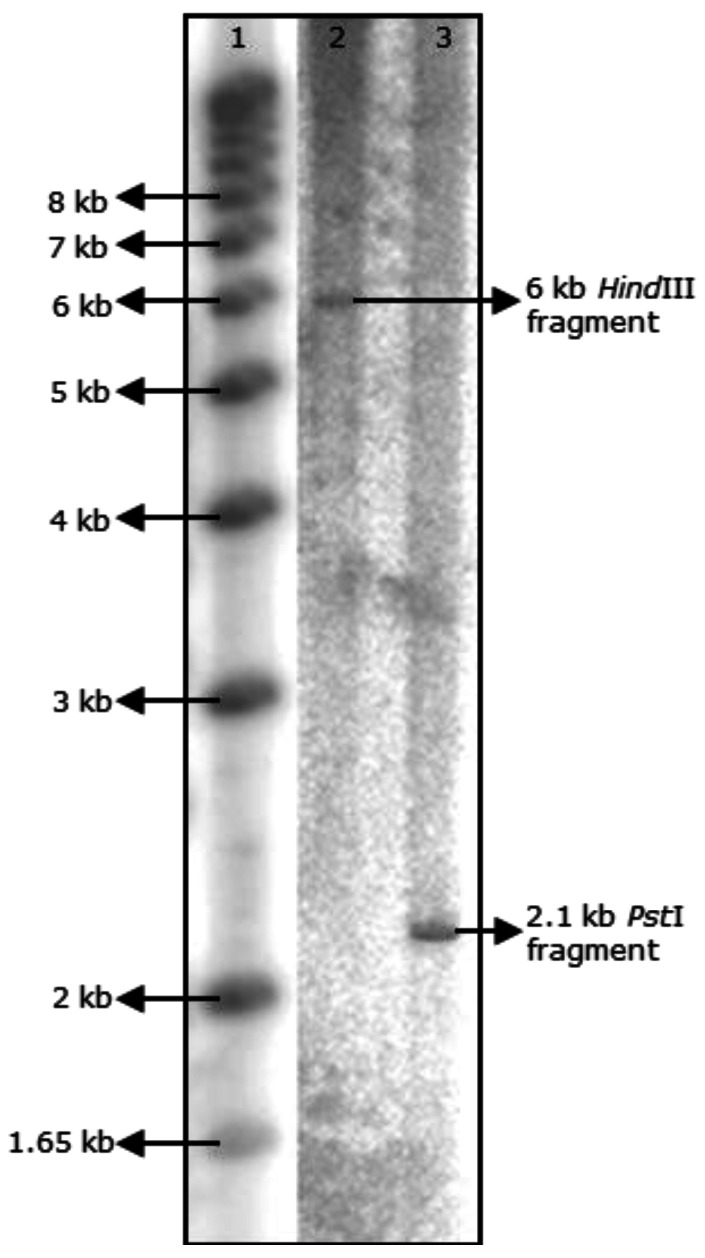
*acuRI* copy number in *A. veronii* MTCC 3249 using southern hybridization. Lane 1: 1 kb Plus DNA marker (Invitrogen); lane 2: *Hind*III digest; and lane 3: *Pst*I digest.

*A. veronii* MTCC 3249 possessed a single copy of the quorum sensing genes suggesting a tight regulation by a singular type of autoinducer (Figure 5). In order to express multiple factors under the control of a single transcriptional factor, there is always a high demand for rRNA transcription. Consistent with this, the strain also possessed 10 copies of the *rrn* operon suggesting strong and efficient transcriptional machinery and a unique ecological strategy adopted by the organism during stages of rapid growth [21]. Given the central role of rRNAs in the regulation of ribosome synthesis, it is conceivable that the number of rRNA operons may dictate the rapidity with which microbes can synthesize ribosomes and respond to favorable changes in growth conditions [23,24]. The copy number of rRNA operons per bacterial genome, which varies from 1 to as many as 15, therefore, reflects an ecological strategy that is characterized either by rapid response to resource input or efficient allocation of resources under constant, slow-growth environments [25,26]. Hence, a detailed investigation into the genes that are regulated by this system would enhance our understanding of the potentially pathogenic mechanisms under its control.

### 3.3. Chemical Characterization of AHLs Produced by *A. veronii* MTCC 3249

**Figure 6.**
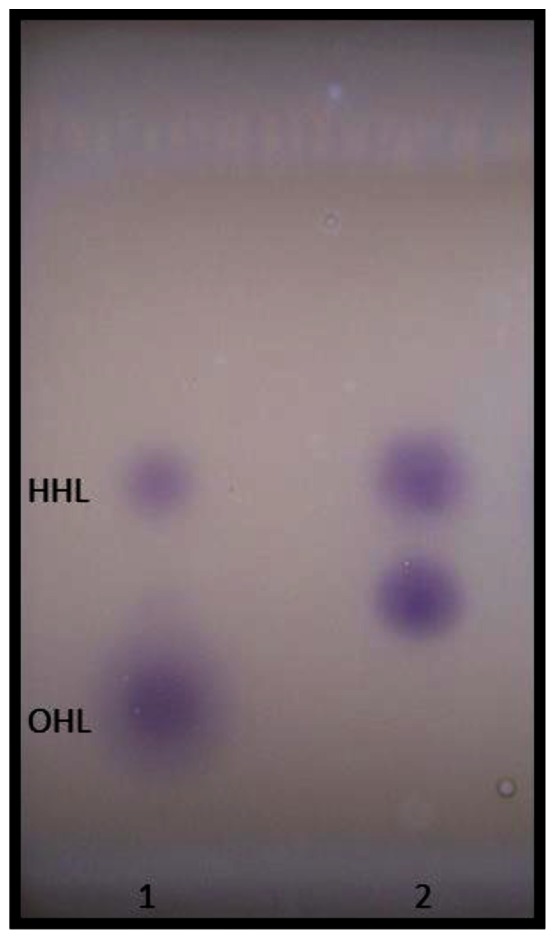
TLC analysis of AHLs produced by *A. veronii* MTCC 3249. Lane 1: mixture of synthetic AHL standards, HHL and OHL; and lane 2: acetonitrile reconstituted extract of AHLs from the culture supernatant.

Using different biosensor strains enabled the detection of a wide range of AHLs produced by *A. veronii*. TLC overlay analysis with *C. violaceum* CV026 revealed a major AHL, which corresponded to the *R_f_* values obtained for synthetic HHL (Figure 6). Additionally, an unknown CV026-positive spot, probably representing a more polar compound, migrated between the synthetic HHL and OHL standards. Although this required further confirmation, our results agree to an extent with previous observations that multiple AHLs are secreted by other members in the genus *Aeromonas.* Studies with *A. hydrophila* and *A. salmonicida* reported two AHLs, BHL (major) and HHL (minor), secreted by the same strain and an additional chemical compound, the identity of which is not yet known [2].

**Figure 7.**
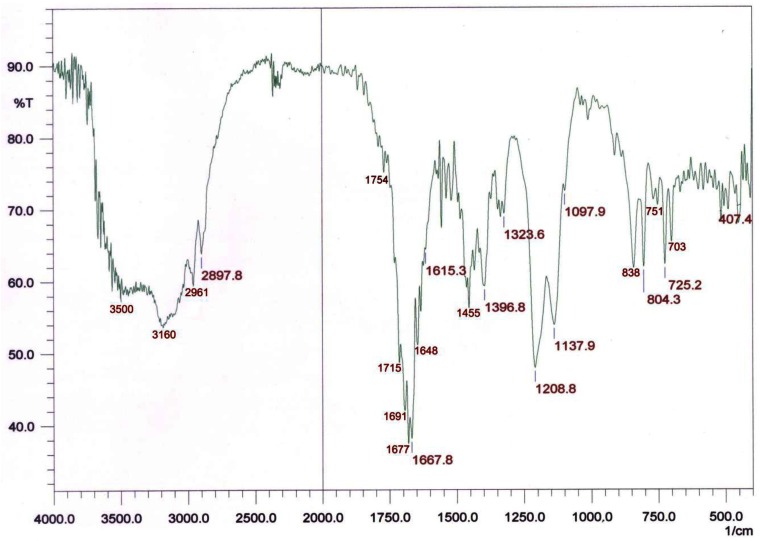
IR spectra of *C. violaceum* CV026 positive HPLC fraction from culture supernatant of *A. veronii* MTCC 3249.

The results of MS, NMR and IR with the purified extracts confirmed this observation and one of the autoinducer compounds was identified to be a carboxyl-acid side chain derivative of *N*-Heptanoyl-L-homoserine lactone. An active HPLC fraction of the AHLs secreted by *A. veronii* MTCC 3249 was further analyzed on MS and NMR (for details see [27]). The IR spectrum peaks at 1754 indicated a gamma -lactone group, as is common in homoserine lactones (Figure 7). The carbonyl peaks at 1,715, 1,677, 1,667, *etc.*, could be attributed to free carboxylic acid or amide groups. The strong absorption at 3,500 indicated OH/NH stretching vibrations.

**Figure 8.**
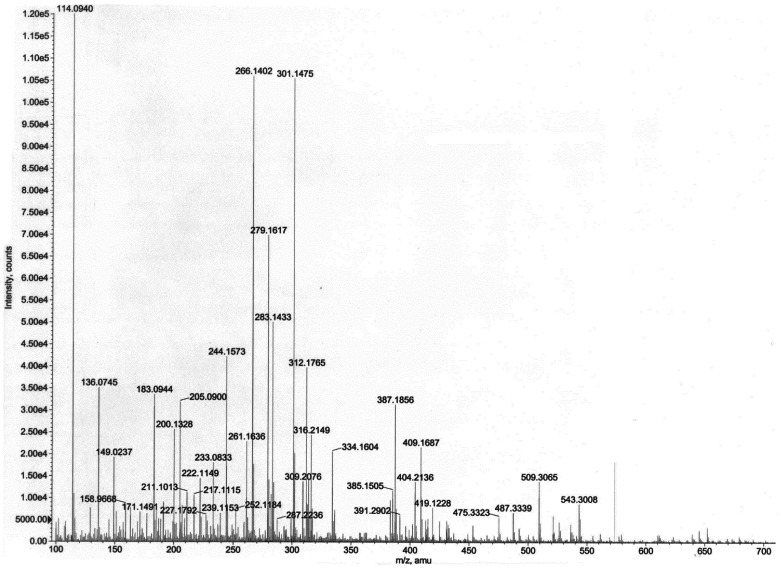
MS/MS scan of *C. violaceum* CV026 positive HPLC fraction from culture supernatant of *A. veronii* MTCC 3249.

**Figure 9.**
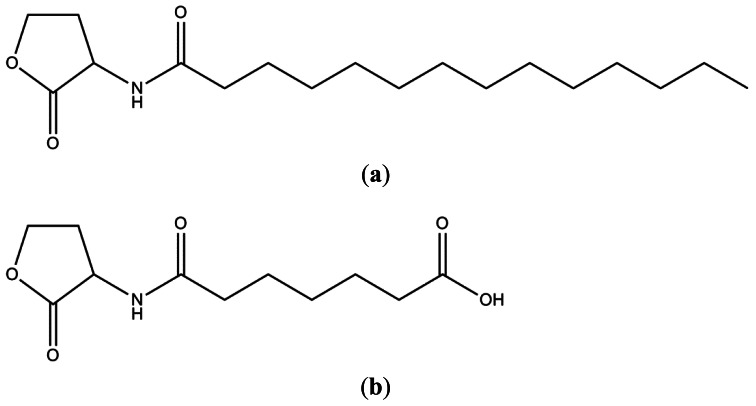
Chemical structures of the AHLs secreted by *A. veronii* MTCC 3249. (**a**) Compound-1: *N*-tetradecanoylhomoserine lactone; (**b**) Compound-2: 6-carboxy-HHL.

The mass spectrum indicated presence of two homoserine lactones of molecular weights 311 and 243, respectively. Thus, the prominent peaks (Figure 8) at *m/z* 645 (2M+Na)^+^, 623 (2M+H)^+^, 334 (M+Na)^+^, 312 (M+H)^+^, 283 (M–CH_3_CH_2_)^+^, 239 (M+H–28–17–28)^+^, 211 (M+H–101)^+^ indicated the presence of a compound of molecular weight 311, while the values at *m/z* 509 (2M+Na)^+^, 487 (2M+H)^+^, 266 (M+Na)^+^, 244 (M+H)^+^, 222 (M–44+Na)^+^, 200 (M–44+H)^+^ indicated presence of a compound having 243 as the molecular weight. Given the homoserine lactone ring, the first compound has C_14_ acid (myrstic acid or tetradecanoic acid) as acyl group (Figure 9), corresponding to an elemental composition of C_18_H_34_NO_3_. The (2M+H)^+^ adduct ions of compound 1 are seen at *m/z* 623 (2M+H)^+^ and 645 (2M+Na)^+^. Other characteristic ions for compound 1 were seen at *m/z* 283 (312-29)^+^ or (M+H–C_2_H_5_)^+^. This loss of terminal ethyl group is known for long chain compounds, such as myrstic acid. Similarly, compound 2 has C_7_ dicarboxylic acid as the acyl moiety. The elemental composition of compound 2 is C_11_H_17_NO_5_. The observed pseudomolecular ions at *m/z* 244 and 266 correspond to C_11_H_18_NO_5_ (M+H)^+^ and C_11_H_17_NO_5_Na, respectively. The (2M+H)^+^ adduct ions of compound 2 are seen at *m/z* 509 (2M+Na)^+^ and 487 (2M+H)^+^ ions. The peak at *m/z* 200 (244–44) or (M+H–CO_2_) is characteristic for carboxylic acids.

The results of MS, NMR and IR confirmed that two types of AHL molecules were secreted by *A. veronii* MTCC 3249 of which one was unique. The production of 6-carboxy-HHL by *A. veronii* is the first report from any bacterium, the only other being from a methanogenic archaeon, *Methanosaeta harundinacea* [28]. In addition, *A. veronii* MTCC 3249 was recently shown to produce several methyl-branched AHLs as well [29]. While the methyl-branched AHLs contained hydroxy acyl chains and unsaturated long acyl chains, our compounds contained neither of these. Although we were unable to detect these methyl-branched AHLs in the HPLC fractions in our study, further chemical analysis of purified fractions from large volumes of culture supernatants is expected to clarify such anomalies.

It is very intriguing that a single strain produces so many different molecules that are potentially under control of a single system. The involvement of such unique types of AHL molecules and the presence of indels in the C-terminal binding domain of AcuR suggests a variant quorum sensing system that is potentially active in *A. veronii* MTCC 3249 [27]. It is interesting to note that this is the first study that reports the involvement of a carboxyl-AHL derivative in the genus *Aeromonas* or for any other bacterial genera.

## 4. Conclusions

We propose the presence of a diverse quorum sensing system in *A. veronii* MTCC 3249 compared to known *luxRI* homologs in *Aeormonas*. The presence of indels in the transcription regulator binding domain, a shorter Lux-box, a novel AHL type and a single copy of the quorum sensing system suggest a tight and highly efficient regulation of mechanisms under its control. The nature of these variations might reflect upon the ecological strategies adopted by the bacterium given the unique habitat and the high cell counts inside the midgut of *Culex quinquefasciatus*. The results presented here reflect a preliminary investigation into the quorum sensing system of *A. veronii* MTCC 3249. However, in order to gain a better understanding of its ecological strategies and pathogenic potential, a detailed investigation into the mechanisms under the control of quorum sensing system in this strain is proposed.

## Figures and Tables

**Table 1.** List of primers used in this study.

Primer Name	Primer Sequence (5′ to 3′)	Remarks
QS-158R	CGC ATT TTC CGC CGT CTC GG	Inverse Sequencing
QS-722F	GGG GGC TAG CAT ATG AAA CAA GAC C	Degenerate
QS-1444R	TTA TTG CAT CAG CTT GGG GAA GTT G	Degenerate
QS-1469F	CAC CAA CTT CCC TAA ACT GAT GCA ATA G	Inverse Sequencing
QS*AcuR*-255R	GGT TCC AGT AGA TGG GCA GCG TC	Inverse
QS*AcuR*-525F	GGT TGT GCG CCT GAG GGA TTC G	Inverse Probe
*AcuI*F	ATG TTG GTT TTC AAA GGA AAA TTG	*A. veronii* specific
*AcuIR*	TTA TAT CTG GGC CGC TAA CTC ATG GGA	*A. veronii* specific
*AcuR*F	ATG AAA CAA GAG CAA CTG TTT GAG TAT	*A. veronii* specific
*AcuR*R	CTA TTG CAT CAG TTT AGG GAA GTT GGT	*A. veronii* specific
